# Excision of rare carotid body tumour without preembolisation: Case report and literature review

**DOI:** 10.1016/j.ijscr.2018.10.029

**Published:** 2018-10-25

**Authors:** S. Shahi, Anupam Raj Upadhyay, Anuj Devkota, Tridip Pantha, Dipendra Gautam, Dhundi Raj Paudel

**Affiliations:** Department of Otorhinolaryngology Head and Neck Surgery,National Academy of Medical Sciences, Bir Hospital, Kathmandu, Nepal

**Keywords:** Carotid body, Paraganglioma, Preembolisation, Excision, Case report

## Abstract

Introduction: Carotid body tumors also known as parganglioma or chemodactomas are one of the rare tumors of head and neck which present as slow growing masses in the neck region. We present a case of 40 years female with painless slow growing mass over left side of her neck for 6 months. Diagnosis was made on basis of clinical history, examination and radiological findings. Tumor was graded as Shamblin grade II. She was managed with excision of the tumor without preoperative embolisation. Intraoperative and postoperative periods were uneventful.

Case presentation: A forty years female presented with left sided painless neck swelling∼5 × 4 cm^2^ over left anterior triangle for 6 months with no history of dysphagia, odynophagia, change in voice, shortness of breath, palpitations, tremors or syncopal attacks. She underwent USG neck and CT angiogram. Based upon the radiological and clinical findings, she was diagnosed asCarotid body tumor. She was managed with excision of the tumor without preembolisation. Her diagnosis was confirmed with histopathology.

Conclusion: Carotid body tumours are rare entities of head and neck region. They are mostly benign in nature. Though mostly bening, increasing size might result in grave complications. Thus, the recommended treatment for carotid body tumors is early excision with or without pre-embolisation. In our case preembolisation was not performed. Though some studies have suggested the use of preoperative embolisation in large sized tumors, more studies are yet required to justify the choice of preembolisation despite the dreaded complications.

## Highlights

• Carotid body tumor is a rare disease entity of head and neck.
• We report a case of carotid body tumor which was treated with excision without preembolisation.
• Early excision of the tumour is mandatory to prevent grave complications.

## 1 Introduction

Historically first described by Von Haller in 1743, Carotid body tumors are also known as paragangliomas, chemodectomas. These are rare paraganglionic tumors commonly found in head and neck. The term paraganglion was coined by Kohn. Clinical presentation of the tumor is an asymptomatic slow growing mass in neck. However, they can produce symptoms due to pressure and local invasion of the surrounding tissue. Most commonly involved are hypoglossal nerve, glossopharyngeal nerve, vagus nerve and sympathetic chain [1,2].

Originally derived from neural crest cells, common site of presentation is the carotid bifurcation. Histologically Carotid body is composed of two types of cells, type I paraganglionic cell also known as chief cell and type II supporting cell also known as sustentacular cell. The characteristic pattern of these tumors are arrangement of type I cells in a pseudoalveolar pattern also known as Zellballen (Cell balls) with finely granular eosinophilic cytoplasm and small oval nuclei. Type II cells have chemoreceptor activity.

They have close resemblance with branchial cysts, tuberculous lymphadenitis, lymphomas, salivary gland tumours, carotid artery aneurysm, metastatic carcinoma or lateral neck masses and thus should be differentiated. But slow growing isolated firm mass in left lateral side of neck with pulsatile nature on palpation makes it easier to delineate it from conditions described above. Clinical history, examination and radiological diagnosis are the keystones to diagnosis and management. Ultrasonogram, CT, MRI are useful radiological tools in diagnosis. Yet Angiography is essential to study about the vascular anatomy [3]. Treatment of choice for carotid body tumors is early excision with or without preembolisation.

## 2 Case presentation

**Fig. 1:**
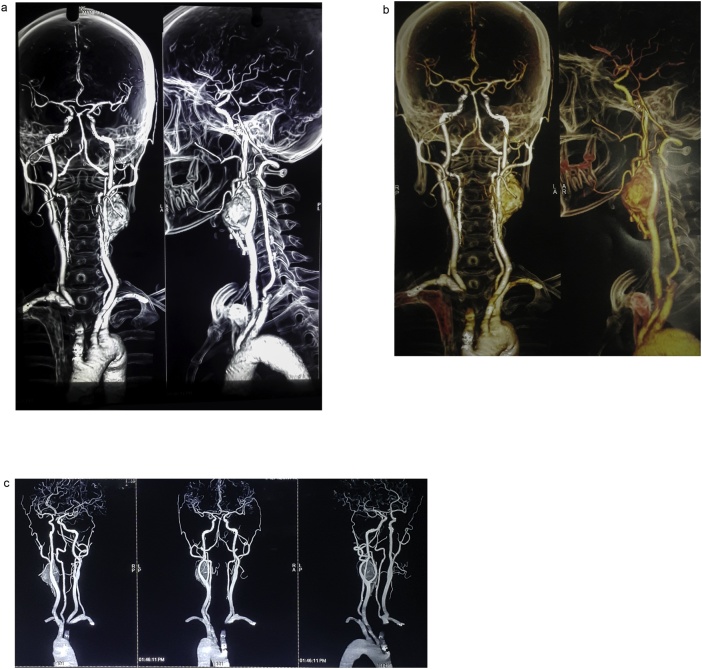
(a–c) CT Angiogram of neck vessels showing heterogenously enhancing lesion at the left carotid bifurcation with splaying of left internal and external carotid arteries.

A forty years female presented with left sided painless neck swelling for 6 months. The swelling was gradually progressive with no aggravating or relieving factors. There was no history of dysphagia, odynophagia or change in voice. She didn’t give any history of shortness of breath, palpitations, tremors or syncopal attacks. There was no history of fever, weight loss or loss of appetite. She denied any history of similar swelling elsewhere in body. There was no history of similar swelling in any family members. On General physical and systemic examination, no abnormality was detected. On examination of the swelling, there was a single globular swelling ∼5 × 4cm^2^ over left anterior triangle region over upper third of anterior border of SCM lateral to hyoid bone. It was non tender, firm with ill defined margins. Surface was smooth and it was pulsatile in nature. Mass was mobile sideways but not vertical (Fonataine’s sign). Fluctuation was absent. There was an absence of carotid bruit. Mass was not bimanually palpable. Mouth opening was adequate, secretions from both the parotid ducts were normal and facial nerve function was intact on both the sides. With the clinical history and findings above she was posed the diagnosis of left carotid body tumor. She underwent USG neck which showed a highly vascular hypoechoeic mass measuring around 3.5 × 3.1 cm^2^ at carotid bifurcation splaying left Internal Carotid Artery and External Carotid Artery. She was then planned for CT angiogram (Fig. 1a–c) which showed well defined heterogeneously enhancing lesion measuring around 3.9 × 3.1 cm^2^ at the carotid bifurcation splaying Internal and External Carotid Arteries. Lesion was perfused by a small twig of proximal left External Carotid Artery. There was mild posterior displacement of left Internal Jugular vein with proximal and distal segments patent. B/L External and Internal Carotid Arteries appeared normal with no mural plaque or canal wall stenosis. The visual branches of External Carotid Artery appeared normal. The features were suggestive of carotid body tumour. Her CBC was within normal limits. Her plasma free metanephrine level was 32.1 pg/ml and Urinary Vanylmandellic Acid 0.8 mg/24 h. CT scan chest and abdomen didn’t show any lesions. Her ECG and Echocardiography didn’t show any abnormal findings. Her chest was clear with a normal pulmonary function test. After the clearance from Anesthesiologist she was planned for excisional biopsy of the tumour at our centre. Intraoperatively we identified 4 × 4×3 cm^3^ firm globular mass over left carotid artery bifurcation partially encasing the External Carotid Artery (Shamblin grade II). Dissection was done along the subadventitial plane. There was a major feeder vessel from External Carotid Artery which was ligated along with small vascular branches around the tumor. Internal Carotid Artery and External Carotid Artery both were preserved. Blood loss was minimal with no injury to adjacent vascular structures. The excised mass was sent for histopathological examination. Postoperative period was uneventful. She was discharged on 7^th^ postoperative day and was followed up after 3 months and showed no signs of recurrence or complications.

**Fig. 2:**
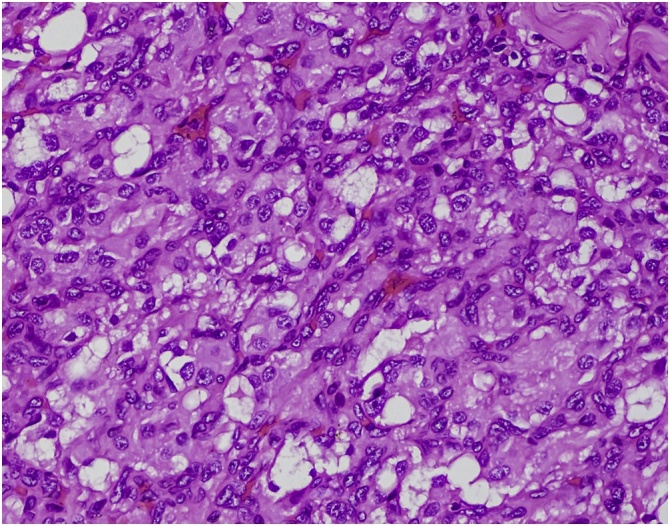
Histopathological study revealing Zellballen pattern of cells with hyperchromatic nuclei and vascular proliferation in between the cells.

The final histopathological reports of the mass revealed Zellballen, alveolar pattern of cells with hyperchromatic nuclei and vascular proliferation in between the cells. Atypical cells were not seen (Fig. 2). The features above were suggestive of paraganglioma.

## 3 Discussion

The case we have presented represents one of the rare entities accounting for 0.06% of head and neck tumors, out of which less than 10% are malignant. Familial accounts for 10% while 31.8% of the familial type are bilateral. Only 6% of the sporadic form are multicentric [4,5].

Mechanisms of Carotid Body tumour is not clear. Hypoxia and genetic factors are considered to be involved in its pathogenesis. According to some studies, disease-induced low partial pressure of the oxygen in the blood and chronic continuous hypoxia in high altitudes (above 1500 m) and chronic intermittent hypoxia due to sleep apnea syndrome could be the stimulus for the hyperplasia and hypertrophy of the carotid glands. Genetic-type amounts to approximately 35%. In individuals with genetic susceptibility, experience of chronic hypoxia might lead to development of tumor at an early age. However family history is not the only governing factor [6]. In our case, the patient came from a low land area with no history of experience to chronic hypoxia and no family history. Malignancy is also evident in some of these neoplasms (<10%) characterised by spread to regional nodes or distant sites (lung, bone, liver, pancreas, thyroid, kidney, breast) [7].

Carotid Body Tumor has been classified by Shamblin Research group into type I (localized type), type II (partially wrapped type) and type III (wrapped type). As the grading goes higher the chances of complications rise. Cranial nerves, Cervical great vessels are at higher risk of injury pre and postoperatively. According to the grading above, it was Shamblin Grade II in our case with no signs of complications.

Recent vascular imaging techniques have also made it easier for better preoperative assessment and planning during surgery [8]. The diagnostic investigation is Carotid angiogram.

Digital Subtraction Angiography (DSA) is the gold standard for diagnosis. However Color Doppler is also done. But DSA is not helpful for information regarding Cranial blood circulation.CT-scan can delineate the relation of tumour with surrounding structures [9].

Definitive management of carotid body tumor is early surgical excision which is equally important to reduce risk of malignancy and perioperative complications. First surgical excision was attempted in 1880 by Reigner but it resulted in postoperative mortality. Maydel in 1886 performed similar procedure and fortunately the patient survived. However hemiplegia and aphasia were the complications. First successful excision was done by Scudder in 1903 [1]. In a study done by Williams MD et al. mortality occurred in 3%, cranial nerve palsies in 13.5%, cranial nerve paresis in 6%, stroke and massive bleeding in 1% each. In the same study, External Carotid Artery ligation was done in 40% [10].

For small tumors excision is simple but for larger tumors and those encasing the arteries, some studies have suggested that division and ligation of External Carotid Artery might be helpful and safe approach for complete excision of the tumors where as ligation of Internal Carotid Artery must be avoided as far as possible to avoid grave complications like stroke. Intraluminal vascular shunts and grafts is used in cases where the vessel intima is damaged [5].

In our case we performed early excision of the tumor without pre embolisation. Preoperative embolisation of the tumor was initially suggested for large tumors with the purpose of reducing blood loss and intraoperative complications but studies now have reported that there is no difference in estimated blood loss, cranial nerve injury, stroke and length of hospital stay [11].

## 4 Conclusion

Early surgical excision of carotid body tumour is the treatment of choice which can be done without preembolisation. However preembolisation is still a matter of controversy. Thus more studies need to be done comparing the outcomes of surgeries with and without pre-embolisation. This case has been reported according to SCARE criteria [12].

## Conflicts of interest

No conflict of interest.

## Funding

No sources of funding.

## Ethical approval

As this is a case report ethical approval has been exempted and written informed consent has been taken from the patient.

## Consent

Written informed consent was obtained from the patient for publication of this case report and accompanying images.

A copy of the written consent is available for review by the Editor-in-Chief of this journal as required.

## Author contribution

Dr.Sudha Shahi- Study concept and design, data collection, literature search, writing paper, final decision to publish.

Dr. Anupam Raj Upadhyay- Literature search, final decision to publish.

Dr. Anuj Devkota- Literature search, final decision to publish.

Dr. Tridip Pantha- Supervised the writing of the manuscript, final decision to publish.

Dr. Dipendra Gautam- Supervised the writing of the manuscript, final decision to publish.

Dr. Dhundi Raj Paudel- Supervised the writing of the manuscript, final decision to publish.

## Registration of research studies

Is a case report.

## Guarantor

Dr. Sudha Shahi

## Provenance and peer review

Not commissioned, externally peer reviewed.
